# Aryl hydrocarbon receptor-microRNA-212/132 axis in human breast cancer suppresses metastasis by targeting SOX4

**DOI:** 10.1186/s12943-015-0443-9

**Published:** 2015-09-17

**Authors:** Hamza Hanieh

**Affiliations:** Laboratory of Physiology, Biological Sciences Department, College of Science, King Faisal University, Faisal Bin Fahd road, Hofuf, 31982 Ahsaa Saudi Arabia

**Keywords:** miR-212/132, Aryl hydrocarbon receptor, Breast cancer, Metastasis

## Abstract

**Background:**

MicroRNAs (miRNAs) are a class of short non-coding RNAs that pave a new avenue for understanding immune responses and cancer progression. Although the miRNAs are involved in breast cancer development, their axis with the transcription factors that show therapeutic potential in breast cancer is largely unknown. Previous studies showed anti-metastatic roles of agonist-activated aryl hydrocarbon receptor (Ahr) in various breast cancer cell lines. Recently, we demonstrated that agonist-activated Ahr induced a highly conserved miRNA cluster, named miR-212/132, in murine cellular immune compartment. Therefore, current study was performed to examine if this miRNA cluster mediates the anti-metastatic properties of Ahr agonists.

**Methods:**

The expression of miR-212/132 cluster and coding genes were examined by real-time PCR, and the protein levels were detected by western blot. The 2,3,7,8-tetrachlorodibenzo-p-dioxin (TCDD) and 3,3′-diindolylmethane (DIM) were used to activate Ahr in MDA-MB-231 and T47D breast cancer cells. Chromatin immunoprecipitation (ChIP) assay was used to identify the binding site(s) for Ahr on miR-212/132 promoter. For prediction of potentially target gene of the miRNA cluster, bioinformatics analysis was carried out, and to test targeting, luciferase activity was quantified. Besides, biological effects of Ahr-miR-212/132 axis were examined *in vitro* by cell migration, expansion and invasion, and examined *in vivo* by orthotopic model of spontaneous metastasis.

**Results:**

The miR-212/132 cluster was transcriptionally activated in MDA-MB-231 and T47D cells by TCDD and DIM, and this activation was regulated by Ahr. A reciprocal correlation was identified between Ahr agonists-induced miR-212/132 and the pro-metastatic SRY-related HMG-box4 (SOX4), and a new specific binding sites for miR-212/132 were identified on the untranslated region (3′UTR) of SOX4. Interestingly, miR-212/132 over-expression showed direct anti-migration, anti-expansion and anti-invasion properties, and an inhibition of the miRNA cluster mitigated the anti-invasive properties of TCDD and DIM. Further *in vivo* studies demonstrated that the Ahr-miR-212/132-SOX4 module was induced by Ahr activation.

**Conclusion:**

Taken together, the findings provide the first evidences of the synergistic anti-metastatic properties of miR-212/132 cluster through suppression of SOX4. Also, current study suggest a new miRNA-based mechanism elucidating the anti-metastatic properties of Ahr agonists, suggesting possibility of using miR-212/132 to control metastasis in breast cancer patients.

**Electronic supplementary material:**

The online version of this article (doi:10.1186/s12943-015-0443-9) contains supplementary material, which is available to authorized users.

## Introduction

Breast cancer is the most common cause of cancer-associated deaths amongst women in developed and developing countries [1]. Metastasis, the spread of a tumor to distant organs, accounts for 90 % of breast cancer patients’ mortality [2]. Important advances have been achieved to understand the complicated process of metastasis, and various molecules have shown promising anti-metastatic properties [3–5]. However, detailed mechanisms remain to be defined.

The microRNAs (miRNAs) are small non-coding RNAs of ∼ 22 nt that regulate gene expression at the post-transcriptional level. These molecules add a new dimension for understanding cancer progression. An increasing paradigm has clearly shown that miRNAs are involved in breast cancer metastasis. For example, miR-10b promotes breast cancer cell invasion and metastasis by targeting syndecan-1 (SDC1) in MDA-MB-231 and MCF-7 cells [6]. The invasion of MDA-MB-231 and BT-20 cells is diminished by over-expression of c-Met-targeting miR-335 [7]. Furthermore, miR-135 and miR-203 reduce tumor growth and metastasis of MD-MB-231 cells to the bones by targeting the runt-related transcription factor 2 (Runx2) [8].

MiR-212 and miR-132 are tandem miRNAs at the same location on chromosome 17 in humans, called miR-212/132 cluster, and they share the same seed sequence and the transcriptional regulatory elements. Extensive studies have revealed important roles of this miRNA cluster in the different body systems, which may suggest potential therapeutic strategy. For example, miR-212/132 cluster is involved in mammary gland development [9, 10], neuronal differentiation and cognitive processes [11–13], cardiac hypertrophy and cardiomyocyte autophagy [14], autoimmune inflammation [15], vasodilatory function and angiogenic responses [16].

In breast cancer, miR-132 suppresses cell proliferation, invasion, migration and metastasis of different breast cancer cells through direct suppression of hematological and neurological expressed 1 (HN1) [17]. Over-expression of this miRNA suppresses proliferation and colony formation of MDA-MB-231 and MCF-7 [18]. Moreover, miR-132 causes expression changes of genes involved in metabolism, DNA damage and cell motility in immortalized fibroblasts co-cultured with epithelial columnar cell hyperplasia (CCH) cells [19]. Although the role of miR-212 has been investigated in different cancer types [20, 21], it has never been investigated alone or in a combination with miR-132 in breast cancer.

The aryl hydrocarbon receptor (Ahr) is an environmentally responsive transcription factor activated by structurally diverse agonists see [22]. It is demonstrated that the Ahr-active omeprazole decreases invasion and metastasis in estrogen receptor (ER)-negative breast cancer cell lines by down-regulation of matrix metalloproteinase-9 (MMP-9) and C-X-C chemokine receptor 4 (CXCR4) [23]. Activation of Ahr by 2,3,7,8-tetrachlorodibenzo-p-dioxin (TCDD) and 6-methyl-1,3,-trichlorodibenzofuran (MCDF) suppresses metastasis of ER-negative breast cancer cells to the lungs [24, 25]. Zhang and colleagues [25] suggest that both TCDD and MCDF induce miR-335 targeting the pro-metastatic mediator SRY-related HMG-box4 (SOX4). However, no more studies were performed to provide more miRNA-based mechanistic explanations.

Activation of Ahr by 3,3′-diindolylmethane (DIM) suppresses breast cancer through repression of CXCR4 and/or CXCL12, and thereby, lowering the invasive and metastatic potential of MDA-MB-231 and MCF-7 cell [26]. In ER-negative breast cancer cell lines, DIM suppresses cell proliferation and motility of MDA-MB-231 by inhibition of phosphorylation of hepatocyte growth factor (HGF) and c-Met at the tyrosines residues [27]. Furthermore, oral treatment of DIM inhibits metastasis of 4T1 cells accompanied by reduced levels of MMP, adhesion molecules, and pro-inflammatory cytokines [28]. Importantly, the underlying mechanisms of the anti-cancer activities of DIM are not simply attributed to the Ahr since DIM is a relatively weak agonist. For example, the DIM inhibits carcinogen-induced mammary tumor growth in Sprague–Dawley rats and this is not concomitant with the hepatic CYP1A1-dependent activity [29]. In addition, no studies have investigated the involvement of miRNAs in the anti-metastatic effect of DIM.

In recent studies, we found that TCDD and 6-formylindolo[3,2-b]carbazole (FICZ) induced the highly conserved miR-212/132 cluster in the murine cellular immune compartment [15, 30]. Therefore, it was hypothesized here that the miR-212/132 cluster may be induced in human breast cancer cells by Ahr agonists, and may contribute to their anti-metastatic properties. Thus, the effects of TCDD and DIM on miR-212/132 expression and metastatic features in human breast cancer cells were investigated. The current results, for the first time, demonstrated that toxic and non-toxic Ahr agonists suppressed breast cancer metastasis through triggering the transcription of SOX4-targeting miR-212/132 cluster. It was further shown that miR-212/132 cluster is a metastasis suppressor in breast cancer cells.

## Results

### TCDD and DIM suppress motility of breast cancer cells in an Ahr-dependent fashion

**Fig. 1:**
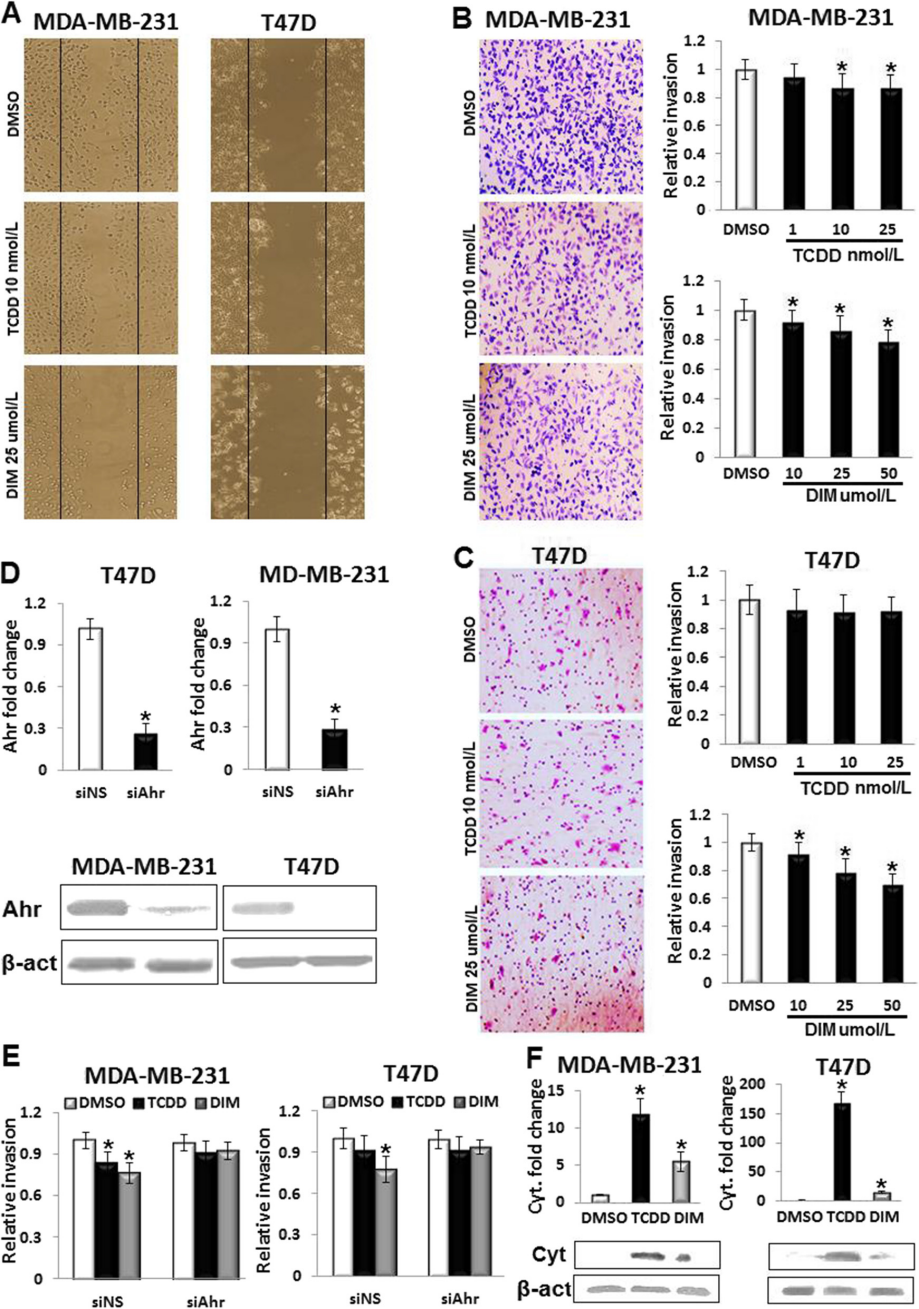
TCDD and DIM inhibit motility of breast cancer cells *in vitro* in an Ahr-dependent fashion. **a** The inhibitory effects of 10 nmol/L TCDD or 25 μmol/L DIM on migration of MDA-MB-231 and expansion of T47D cells were examined by wound healing assay. **b** Cell invasion of MDA-MB-231 was suppressed by TCDD (10–25 nmol/L) and DIM (10–50 μmol/L) in the Boyden chamber assay. **c** DIM (10–25 μmol/L), but not TCDD, inhibited the invasion of T47D cells. **d** Efficiency of Ahr knockdown by siAhr compared with siNS was confirmed by real-time PCR and western blot. **e** siAhr abrogated the inhibitory effects of TCDD and DIM on the invasion of MDA-MB-231 and T47D cells in the Boyden chamber. **f** Activation of Ahr by TCDD (10 nmol/L) and DIM (25 μmol/L) was confirmed by quantification of CYP1A1 gene expression by real-time PCR and western blot. Data are shown as mean ± SD of three independent experiments performed in triplicates. **b**, **c** and **f** *; *P* < 0.05, significantly different from DMSO-treated control. **d** *; *P* < 0.05, significantly different from siNS-transfected control. **e** significantly different from DMSO- and siNS-treated control

A wound healing assay was used to examine the effects of TCDD and DIM on migration of MDA-MB-231 and proliferation-based expansion of T47D. The TCDD (10 nmol/L) and DIM (25 μmol/L) suppressed cell migration of MDA-MB-231, whereas the expansion of T47D was inhibited by DIM only (Fig. 1a). Inhibition of Ahr using silencing RNA (siAhr) blocked the effect of TCDD on the migration of MDA-MB-231 cells, while partially inhibited the anti-expansion effect of DIM on both cell lines (Additional file 1: Figure S1), suggesting an Ahr-independent effects of DIM. No significant effects were observed on the proliferation of MDA-MB-231 cells with TCDD (1–25 nmolL) or DIM (10–50 μmol/L) treatments (Additional file 1: Figure S2A). The DIM, but not TCDD, suppressed the proliferation of T47D and adhesion of T47D and MD-MB-231 in a concentration-dependent fashion (Additional file 1: Figure S2B and C). The inhibitory effects of DIM on proliferation and adhesion of T47D might have contributed to the suppressive effect of DIM on cell expansion in wound healing. To further assess the effects of Ahr agonists on motility of breast cancer cells, cell invasion was examined by Boyden chamber assay. The results in Fig. 1b and c show that TCDD (10–25 nmol/L) and DIM (10–50 μmol/L) significantly decreased cell invasion of MD-MB-231, whereas the invasion of T47D cells was decreased by DIM only (10–50 μmol/L).

The role of Ahr in mediating the inhibitory effects of TCDD and DIM on the invasion of breast cancer cells was investigated by inhibition of Ahr using silencing RNA (siAhr). Transfection of siAhr drastically decreased Ahr gene expression compared with non-specific nucleotides (siNS)-transfected controls of MDA-MB-231 and T47D (Fig. 1d). Knockdown of Ahr abrogated the inhibitory effects of TCDD (10 nmol/L) and DIM (25 μmol/L) on invasion of MDA-MB-231 and T47D cells (Fig. 1e), showing that Ahr mediated the agonists-suppressed invasion of breast cancer cells. Activation of Ahr by TCDD and DIM was confirmed by the quantification of CYP1A1 gene expression (Fig. 1f).

### Agonist-activated Ahr regulates miR-212/132 expression in breast cancer cells

**Fig. 2:**
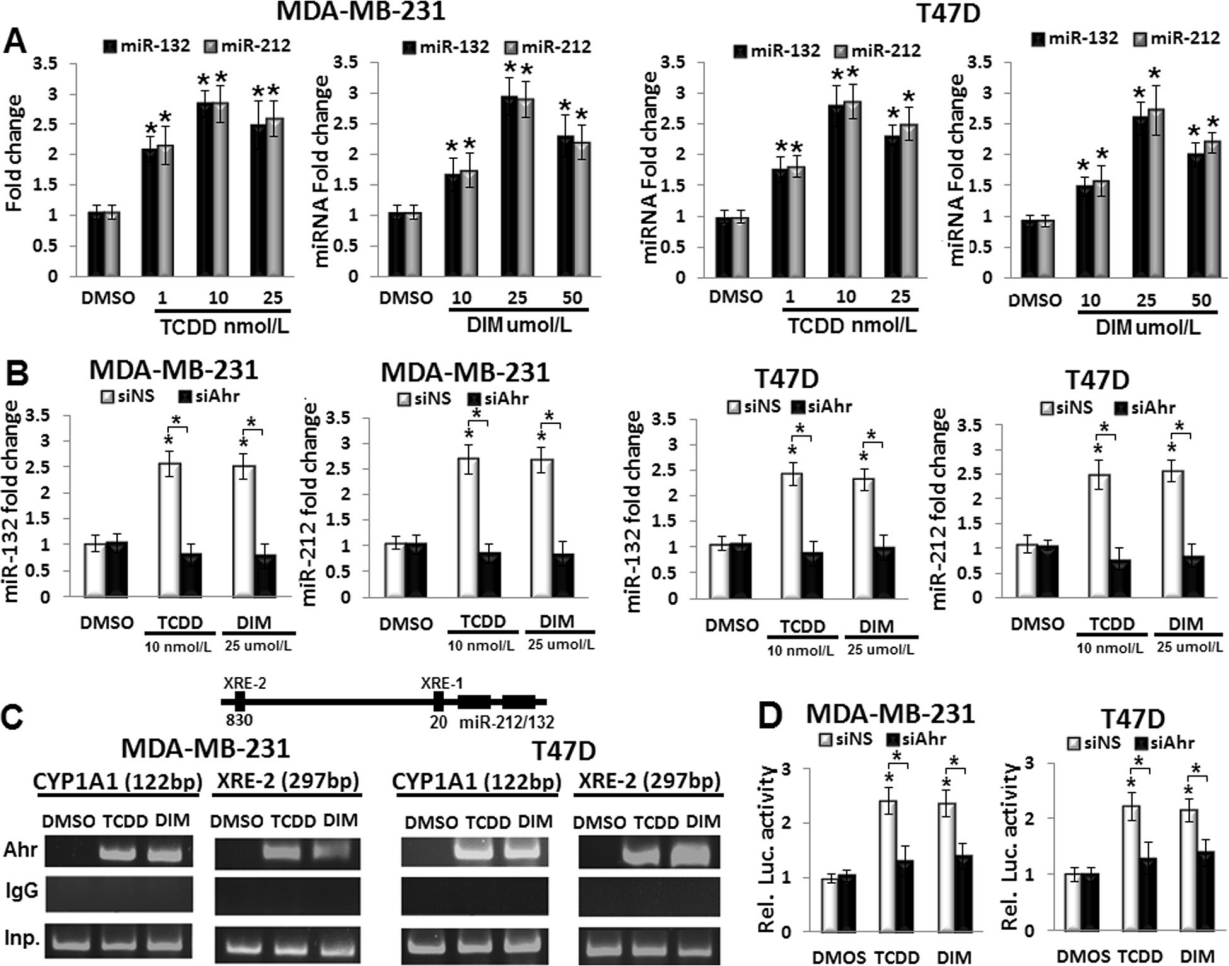
TCDD and DIM induce miR-212/132 cluster in breast cancer cells in an Ahr-dependent fashion. **a** TCDD (1–25 nmol/L) and DIM (10–50 μmol/L) induced miR-212/132 cluster in MDA-MB-231 and T47D cells, miRNA expression was quantified by real-time PCR. **b** siAhr blocked the 10 nmol/L TCDD- and 25 μmol/L DIM-induced miR-212/132 expression in MDA-MB-231 and T47D cells compared with the siNS-transfected control. **c** ChIP assay analysis of Ahr binding activity on XRE box on upstream sequence of miR-212/132 gene. The breast cancer cells were treated with 10 nmol/L TCDD or 25 μmol/L DIM for 24 h. Presented is XRE-2 that showed interaction with Ahr. **d** siAhr suppressed the luciferase activity of miR-212/132 promoter reporter. Data are shown as mean ± SD of three independent experiments performed in triplicates. **a** *P < 0.05, significantly different from DMSO-treated control. **b** and **d** *P < 0.05, significantly different from DMSO-treated control or Ahr agonist- and siAhr-treatments

To examine the hypothesis of Ahr-miR-212/132 axis in breast cancer cells, the expression of miR-212/132 cluster was measured by real-time PCR. Both TCDD (10 nmol/L) and DIM (25 μmol/L) induced the miRNAs cluster in MDA-MB-231 and T47D in 24 h after treatment (Additional file 1: Figure S3A). However, the expression of the miRNA cluster peaked with less standard deviation at 48 h after TCDD (1–25 nmol/L) and DIM (10–50 μmol/L) treatments in both cell lines (Fig. 2a). To support these findings, two more Ahr-specific agonists were used to examine their effects on the miR-212/132 cluster expression. Activation of Ahr by 2-(1′H-indole-3′-carbonyl)-thiazole-4-carboxylic acid methyl ester (ITE; 100 nmol/L) and 3-methylcholanthrene (3MC; 1 μmol/L) induced the expression of miRNA cluster in MDA-MB-231 and T47D at 48 h after treatment (Additional file 1: Figure S3B). These results suggested that the agonist-activated Ahr was involved in up-regulation of miR-212/132 in both breast cancer cell lines.

To investigate whether Ahr was directly involved in miR-212/132 expression, first, Ahr was inhibited by RNA interference. Results illustrated in Fig. 2b show that siAhr blocked the Ahr agonist-induced miR-212/132 cluster. To further test a direct regulatory role of Ahr, 1 kb of miR-212/132 promoter was analyzed for the xenobiotic responsive elements (XRE) using transcription factor prediction software. i.e., Promo V3.0.2 [31]. Binding activity of Ahr to the two xenobiotic responsive elements (XRE) located within 1 kb in the promoter of miR-212/132 gene were examined by Chromatin immunoprecipitation (ChIP) assay. The Ahr physically bound to the XRE-2 located at 830 bp from miR-212/132 transcription site (Fig. 2c). Binding of Ahr to the XRE box of a well-known responsive gene CYP1A1 was used as positive control. Finally, luciferase activity was quantified in breast cancer cells co-transfected with miR-212/132 promoter reporter and siAhr. Knockdown of Ahr in Ahr agonists-treated cells significantly inhibited the luciferase activity driven by miR-212/132 promoter (Fig. 2d). Collectively, the results demonstrated for the first time that Ahr directly regulated miR-212/132 transcription by functional binding on miR-212/132 promoter.

### MiR-212/132 has a direct inhibitory role on motility of breast cancer cells

**Fig. 3:**
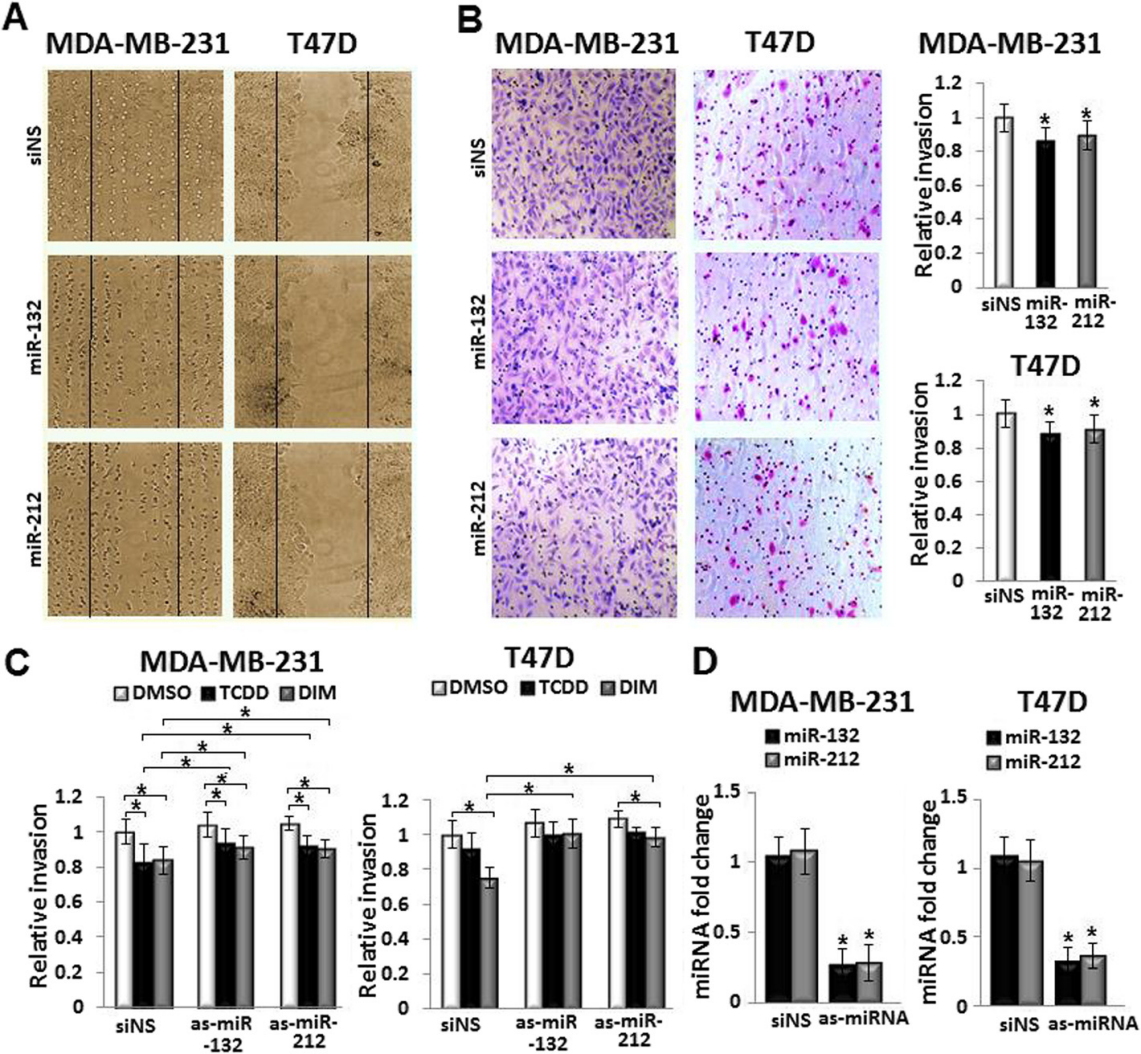
MiR-212/132 cluster has a direct inhibitory role on motility of breast cancer cells *in vitro*. The role of miR-212/132 cluster on migration of MDA-MB-231, expansion of T47D and invasion of breast cancer cells were examined by the transfection of miRNA mimics, miR-132 and miR-212, or antisense, as-miR-132, as-miR-212. **a** Over-expression of the miR-212/132 cluster by mimics suppressed the migration of MDA-MB-231 and expansion of T47D cells in wound healing assay compared with siNS-transfected control. **b** Mimics of the miR-212/132 cluster suppressed the invasion of MDA-MB-231 and T47D cells in the Boyden chamber assay. **c** Inhibition of miR-212/132 cluster by antisense partially blocked the inhibitory effects of TCDD and DIM on invasion of MDA-MB-231 and T47D cells. **d** Efficiency of miR-212/132 knockdown by antisense compared with siNS was confirmed by real-time PCR. Data are shown as mean ± SD of three independent experiments performed in triplicates. **b** and **d** *P < 0.05, significantly different from siNS-transfected control. **c** *P < 0.05, significantly different as shown by lines

To study whether the miR-212/132 cluster has a direct role on motility of breast cancer cells, the cluster mimics were transfected separately into MDA-MB-231 and T47D cells. Over-expression of miR-132 and miR-212 showed inhibitory effects on migration of MDA-MB-231 and expansion of T47D cells in wound healing assay, and invasion in Boyden chamber in both cell lines compared with siNS-transfected controls (Fig. 3a and b). Consistently, inhibition of the miRNA cluster by transfection of antisense (as-) into the TCDD- or DIM-treated breast cancer cells mitigated the agonists’ inhibitory effects on invasion of MDA-MB-231 and T47D (Fig. 3c), suggesting a direct inhibitory role of miR-212/132 on motility of MDA-MB-231 and T47D cells. Efficiency of miR-212/132 inhibition by antisense was confirmed by real-time-PCR as shown in Fig. 3d.

### TCDD and DIM down-regulate SOX4 in breast cancer cells by triggering Ahr-miR-212/132 axis

**Fig. 4:**
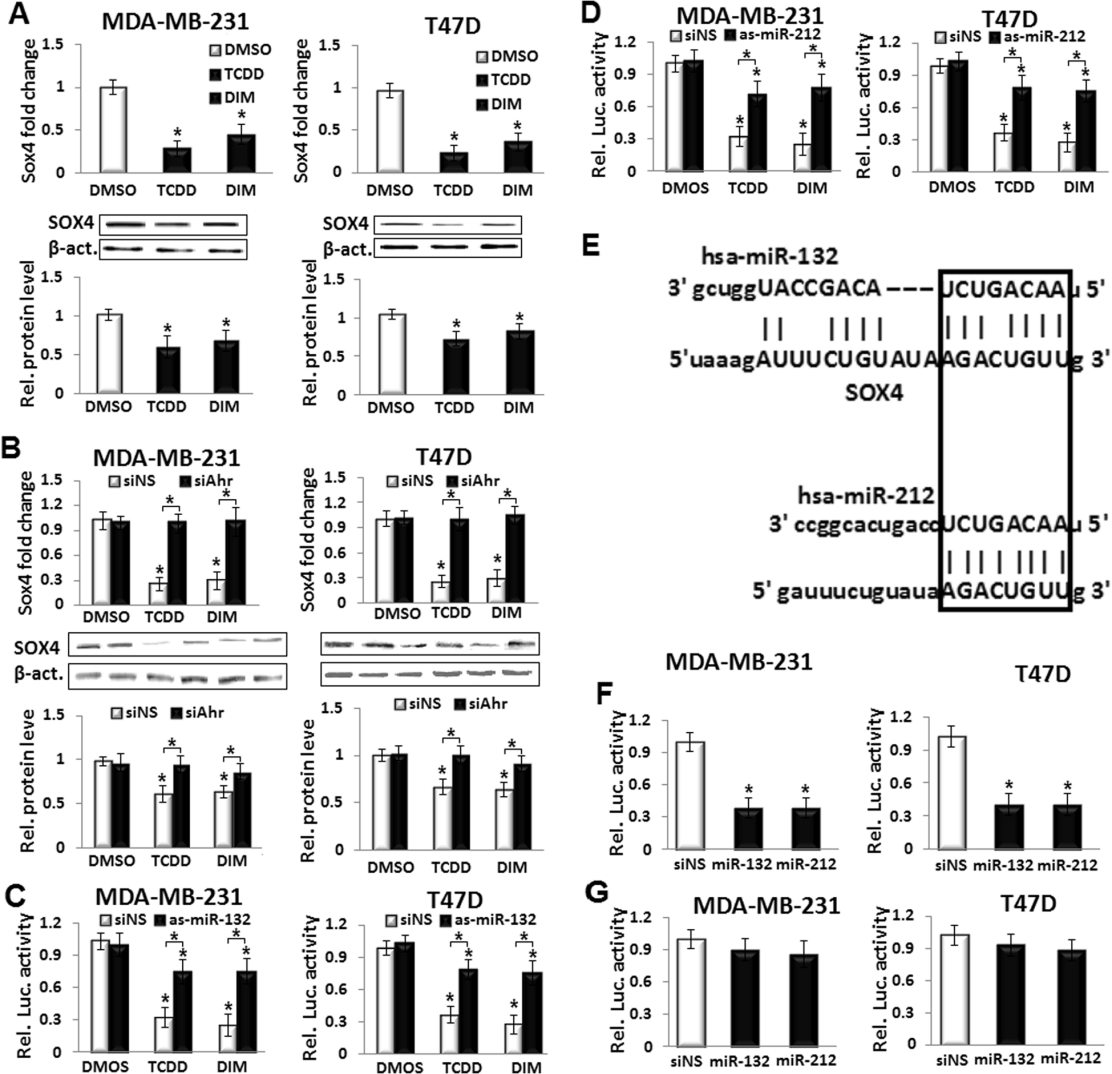
TCDD and DIM suppress the pro-metastatic factor SOX4 by inducing the miR-212/132 cluster. **a** 10 nmol/L TCDD and 25 μmol/L DIM suppressed SOX4 gene expression as quantified by real-time PCR and western blot. **b** Knockdown of Ahr by siAhr blocked the inhibitory effects of TCDD and DIM on SOX4 compared with siNS-transfected MDA-MB-231 and T47D cells. **c** and **d** Co-transfection of 3′UTR-SOX4-luc and miRNA antisense, as-miR-132 or as-miR-212, mitigated the inhibitory effects of TCDD and DIM on the luciferase activity in MDA-MB-231 and T47D. **e** SOX4 is a potential target gene of the miR-212/132 cluster; complementary binding site of miR-212/132 on the SOX4 3′UTR. **f** Co-transfection of 3′UTR-SOX4-luc and miRNA mimics, miR-132 or miR-212, suppressed the luciferase activity in MDA-MB-231 and T47D. **g** MiRNA mimics did not decrease the luciferase activity when co-transfected with 3′UTR-SOX4-luc that contains mutated binding sites for miR-212/132. Data are shown as mean ± SD of three independent experiments performed in triplicates. **a** *P < 0.05, significantly different from DMSO-treated control. **b**–**d** *P < 0.05, significantly different from the corresponding DMSO- and siNS-treated control or Ahr agonist- and miRNA antisense treatments. **f** and **g** *P < 0.05, significantly different from siNS-transfected control

The effects of TCDD and DIM on gene expression of the pro-metastatic factor SOX4 were examined by real-time PCR and western blot. Both TCDD and DIM significantly down-regulated SOX4 gene expression in MDA-MB-231 and T47D cells (Fig. 4a). Conversely, inhibition of Ahr by siAhr restored the Ahr agonists-suppressed SOX4 in both cell lines (Fig. 4b), suggesting that TCDD and DIM down-regulated SOX4 in an Ahr-dependent fashion. In addition, the role of SOX4 in mediating the inhibitory effects of Ahr agonists on migration, expansion and invasion of breast cancer cells was confirmed by over-expression of SOX4. The TCDD- and DIM-mediated inhibition of breast cancer cell migration, expansion and invasion was moderately abrogated by SOX4 over-expression (Additional file 1: Figure S4A and B).

The interaction of TCDD and DIM with SOX4 was further analyzed by measuring the luciferase activity. The MDA-MB-231 and T47D cells were transfected with 3′UTR-SOX4-luc construct, which contains the candidate binding sites for miR-212/132 cluster (MiRaNda v.21) [32]. Activation of Ahr by TCDD and DIM decreased the luciferase activities significantly, and these effects were reversed by co-transfection with as-miR-132 (Fig. 4c) and as-miR-212 (Fig. 4d), suggesting a regulatory role of the miRNA cluster on SOX4 gene expression.

### SOX4 is a new target gene of miR-212/132 cluster in breast cancer cells

An application of miRNA target scan algorithm, i.e., MiRaNda [32], predicted that SOX4 is a candidate target gene of the miR-212/132 cluster (Fig. 4e). To examine this prediction, MDA-MB-231 and T47D cells were first transfected with miR-212/132 mimics. Over-expression of miR-212/132 in both cell lines decreased SOX4 mRNA (Additional file 1: Figure S5A and B). The cells were also co-transfected with 3′UTR-SOX4-luc construct and miR-212/132 mimics. As shown in Fig. 4f, over-expression of miR-212/132 significantly decreased the luciferase activity. These effects were abrogated when the 3′UTR-SOX4-luc was replaced with a reporter contains a mutated sequence where miR-212/132 seed sequence binds (Fig. 4g). These results identified SOX4 as a new target gene of the miR-212/132 cluster in MDA-MB-231 and T47D cells.

### The suppressive effects of TCDD and DIM on metastasis of breast cancer cells is concomitant with higher miR-212/132 expression

**Fig. 5:**
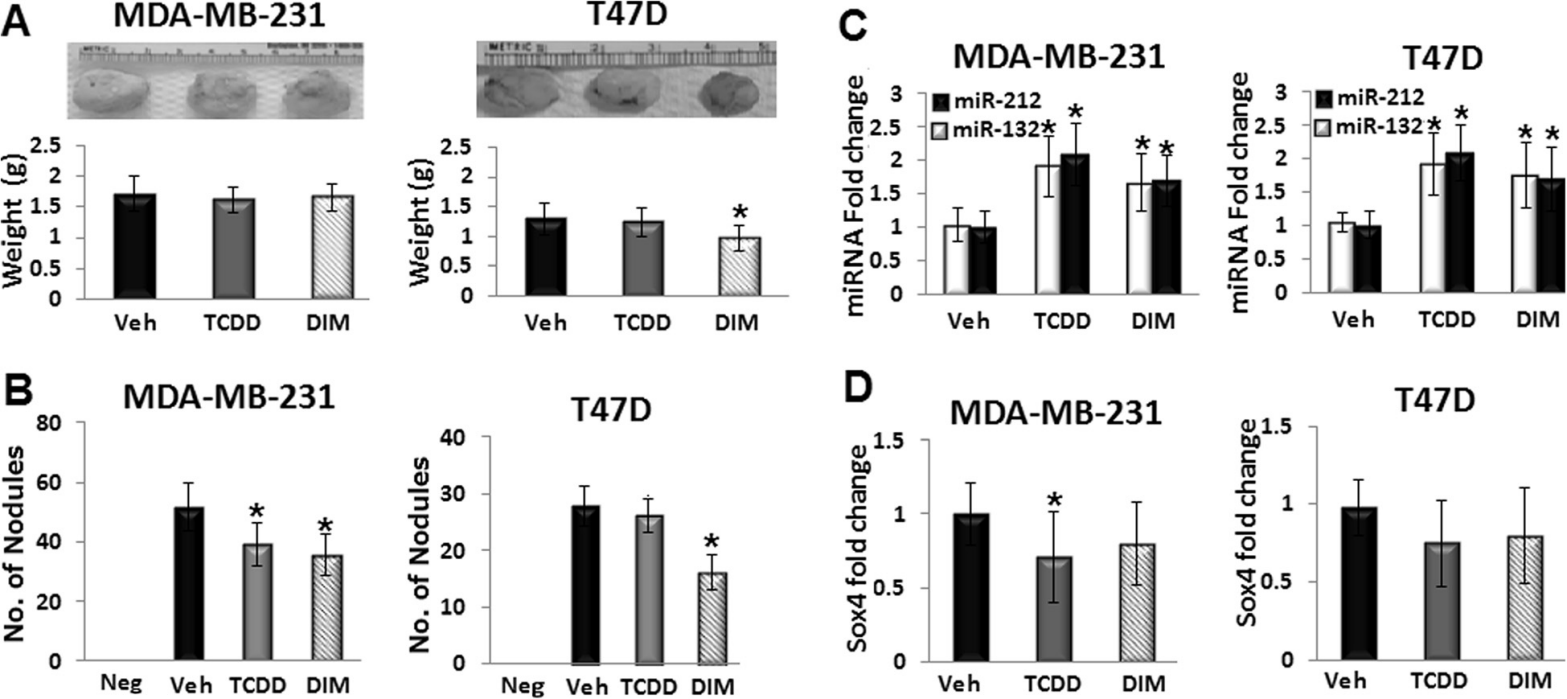
TCDD and DIM suppress metastasis of breast cancer cells to the lungs and induce miR-212/132 *in vivo*. MDA-MB-231 or T47D were injected in the fat pad of the second nipple of athymic nude mice. TCDD (25 μg/kg/day), DIM (50 mg/kg/day), or corn oil (Veh) were given orally for 10 days. Non-transplanted (Neg) mice were used as negative controls. **a** DIM treatment decreased the weight of T47D primary tumor that formed at the breast cancer cell injection site. **b** TCDD and DIM treatments decreased the number of pulmonary nodules in MDA-MB-231-injected mice, while the number of the nodules in T47D-injected mice was decreased with DIM treatment. **c** TCDD and DIM induced the miR-212/132 expression in pulmonary nodules of MDA-MB-231- or T47D-injected mice. **d** TCDD treatment suppressed SOX4 mRNA in the pulmonary nodules of MDA-MB-231-injected mice. Data are shown as mean ± SD of three independent experiments. *n* = 8 in MDA-MB-231-injected mice. **a**–**d** *P < 0.05, significantly different from Veh-treated control

The effects of Ahr agonists on Ahr-miR-212/132-SOX4 module *in vivo* were tested using an established orthotopic model of tumor growth and spontaneous metastasis. Injection of MDA-MB-231 into the nipple fat pad of athymic mice, and to a lesser extent T47D, resulted in formation of primary tumor at the injection site and pulmonary nodules that were adequate to represent spontaneous metastasis (Additional file 1: Figure S6). The TCDD at 25 μg/kg/day or DIM at 50 mg/kg/day was given orally for 10 days starting from the day of breast cancer cell injection. The Ahr agonists treatments did not show statistical significance on body and lung weights compared with corn oil (Veh)-treated controls (data not shown). Consistent with the *in vitro* results, treatment of T47D-injected mice with DIM decreased the weight of the primary tumor that formed at the injection site in 4 weeks (Fig. 5a). Interestingly, the number of MDA-MB-231 pulmonary nodules was less in Ahr agonists-treated mice compared with Veh-treated control, whereas the number of T47D nodules decreased with DIM treatment only (Fig. 5b).

To test whether Ahr agonists induce miR-212/132-SOX4 module *in vivo*, miRNA cluster and SOX4 mRNA were quantified in the pulmonary nodules. TCDD, and to a lesser extent DIM, significantly induced the expression of miR-212/132 cluster in the isolated nodules of MDA-MB-231- or T47D-injected mice (Fig. 5c). The SOX4 mRNA was significantly down-regulated only in MDA-MB-231-injected mice with the TCDD treatment. Although mean values of SOX4 mRNA were less in other treatment groups compared with Veh-treated controls, they did not reach the level of significance (*P* < 0.05) as shown in Fig. 5d.

## Discussion

The miRNAs are small non-coding RNAs that regulate gene expression by complementary binding on the 3′UTR of the target mRNA. Accumulating evidences have demonstrated that miRNAs are involved in the progression or inhibition of different tumors including breast cancer [33, 34]. In breast cancer, metastasis is a complicated process that contributes to a high mortality rate among other cancer patients [2]. It has been indicated that miRNAs modulate metastasis of breast cancer cells to different organs of the body [35, 36]. However, their interaction with the transcription factors that play prominent roles in breast cancer metastasis is rarely investigated.

The Ahr has been extensively studied in different tumors and cell lines of breast cancer, and now it is clear that Ahr plays critical roles in modulating tumor progression [37, 38]. Current study showed that TCDD inhibited invasion of MDA-MB-231 cells *in vitro*, whereas DIM showed inhibitory effects on both cell lines. These results were in a line with previous studies showed that TCDD inhibited cell invasion in ER-negative breast cancer cell lines in an Ahr-dependent fashion [25, 39]. The inhibitory effects of DIM on cell invasion in ER-negative and ER-positive cell line were also reported [40–42]. The *in vivo* results showed that DIM, but not TCDD, decreased the primary tumor weight that formed at the T47D injection site. In contrast, previous studies showed that TCDD and TCDD-related compounds inhibited mammary tumor growth [43, 44]. These discrepancies were likely attributed to the rodent model used, doses of Ahr agonists and the number of these doses.

In previous studies, we demonstrated that activation of Ahr by TCDD and FICZ induced the highly conserved miR-212/132 cluster in murine cellular immune compartment, and supporting results were obtained in Ahr^−/−^ mice [15, 30, 45]. Therefore, it was predicted in the current study that Ahr agonists induce miR-212/132 in human breast cancer cell lines, and these miRNAs may contribute to the anti-tumor properties of these agonists. Activation of Ahr by TCDD and DIM suppressed migration of MDA-MB-231 and proliferation-based expansion of T47D cells, and knockdown of Ahr reversed these effects. Inhibition of miR-212/132 in Ahr-agonist treated cells mitigated the anti-invasive effects of the agonists, and transfection with mimics showed supporting results, suggesting that miR-212/132 cluster mediated, at least partially, the anti-invasive effects of TCDD and DIM. Also, these results indicated an involvement of other molecules, probably other miRNAs, in the anti-invasive effects of Ahr agonists.

MiR-212 and miR-132 are tandem miRNAs located in an intergenic region on chromosome 17 in humans, and they share the same seed sequence AACAGUCU. Analysis of the regulatory elements of the miR-212/132 gene using online software, i.e., Promo V3.0.2 [31], revealed the presence of two XRE boxes (GCGTG) located within 1 kb relative to the transcription site. A direct regulatory role of Ahr on the miR-212/132 gene by association with XRE boxes was tested by ChIP assay, and confirmed by luciferase activity. The results identified a functional XRE box, located at 830 bp from the transcription site, on which Ahr was bound. Together, these results confirmed for the first time that Ahr directly regulated the transcription of miR-212/132 gene.

The SOX4 transcription factor belongs to the SOX (SRY-related HMG-box) family that is involved in embryonic development and cell fate. It is demonstrated that an inhibition of SOX4 is associated with a decreased invasion and metastasis of breast cancer cells [46]. It is also shown that TCDD and MCDF down-regulate SOX4 in MDA-MB-231 and BT474 by inducing miR-335 [25]. These effects are abrogated by Ahr knockdown and mitigated by miR-335 antisense in Ahr agonists-treated cells. These results are in complement with those obtained in the current study. Both TCDD and DIM reduced the luciferase activity of the 3′UTR-SOX4 construct that contains the predicted binding sites for the miR-212/132 cluster, and mutation in these sites restored the luciferase activity. Also, co-transfection of the 3′UTR-SOX4-luc and miR-212/132 mimics or miRNA mimics alone showed supporting results. Taken together, the results identified SOX4 as a new target gene of the miR-212/132 cluster in human breast cancer cells. Notably, the effects of Ahr agonists on SOX4 were repealed by Ahr knockdown and partially reversed by miR-212/132 antisense in Ahr agonist-treated cells, suggesting an involvement of other molecules, i.e., miRNAs, in the regulatory role of Ahr agonists on SOX4.

The involvement of Ahr-miR-212/132-SOX4 module in the anti-metastatic properties of TCDD and DIM on MDA-MB-231 and T47D were investigated in the pulmonary nodules using a demonstrated model of spontaneous metastasis. Previous studies showed that oral treatment of TCDD and DIM in breast cancer cells-injected mice resulted in a reduction in the pulmonary tumor nodules [24, 28]. These results were in line with those obtained in the current study using MDA-MB-231 and T47D cells. Consistent with the *in vitro* results, TCDD induced a reciprocal correlation between miR-212/132 and SOX4 *in vivo*, which may explain, at least partially, the anti-metastatic properties. The DIM treatment induced miR-212/132 *in vivo*, to a lesser extent compared with TCDD, and mean value of SOX4 did not reach the significance level (*P* < 0.05). These differences may be attributed to the agonists’ chemical structure. TCDD contains four chlorine residues that give this agonist stability and longer half-life, which attribute to strong and sustained Ahr activation see [22].

Previous studies have suggested different mechanistic explanations for the *in vitro* anti-invasive and the *in vivo* anti-metastatic properties of Ahr agonists. For example, TCDD and MCDF induce SOX4-targeting miR-335 in MDA-MB-231 and BT474 cells *in vitro* [25]. It has been also suggested that TCDD disrupt the CXCR4/CXCL12 axis in an *in vitro* chemotaxis assay [47]. Oral administration of DIM suppresses 4T1 metastasis to the lungs by inhibition of two MMPs, adhesion molecules, and pro-inflammatory cytokines [28]. Furthermore, different Ahr agonists show anti-estrogen effects by inhibitory cross talk between Ahr and ER in ER-positive breast cancer cells [48, 49]. In the current study, over-expression of miR-212/132 showed anti-invasive properties, and inhibition of Ahr agonist-induced miRNA cluster abrogated the agonists’ anti-invasive properties in MDA-MB-231 and T47D. These results suggest a new mechanism through which miR-212/132 mediate the anti-metastatic properties of TCDD and DIM by targeting the pro-metastatic factor SOX4.

It has been shown that the constitutively active Ahr enhances growth and motility of breast cancer cells by different mechanisms such as transcription of breast cancer gene 1 (BRCA1) oncogene and CYP1B1, and activation of epiregulin and Wnt signaling pathway see [50]. Therefore, inhibition of certain endogenous Ahr agonists has suppressive effects on breast cancer progression. This does not reflect contradiction with the inhibitory properties of Ahr agonists, as agonists may force Ahr to do different functions. For example, while endogenous ligands enhance cancer by transcription of BRCA1 oncogene, activation of Ahr by exogenous agonists suppress BRCA1 gene expression [51].

## Conclusion

Taken together, the results not only provide a new miRNA-based mechanism to understand the anti-metastatic properties of Ahr agonists, but also provide the first evidence of the synergistic anti-metastatic properties of the members of miR-212/132 cluster in human breast cancer cells, opening intriguing possibilities of using this miRNA cluster as an innovative therapeutic strategy for breast cancer.

## Materials and methods

### Cell culture

The human breast cancer cell lines MDA-MB-231 and T47D were obtained from American Type Culture Collection (ATCC; Manassas, VA). The cells were maintained in a complete medium contining Dulbecco’s modified Eagle’s medium (DMEM)/Ham’s F12 nutrient mixture (F-12) at 1:1 (Sigma-Aldrich, St Louis, MO) supplemented with 10 % FBS and 1× of antibiotic antimycotic solution (Gibco, Rockville, MD). Cells were incubated in a humidified atmosphere with 5 % CO_2_ at 37 °C.

### Wound healing and invasion assays

Cells were seeded in 6-wells plate in a complete medium for 24 h to attach. The cells were then treated with DMSO, TCDD (Accustandard, New Haven, CT) or DIM (Sigma-aldrich) for 24 h in 3 % charcoal-stripped FBS (Sigma-Aldrich) DMEM/F-12. When cells reached 60–80 % confluent, a scratch was made at the axis of the well using pipette tip, washed, and then treated again with DMSO or Ahr agonists for another 24 h. The T47D medium was supplemented with 10 nmol/L β-estradiol (E2; Santa Cruz Biotechnology, Santa Cruz, CA). A 24-wells matrigel coated Boyden chamber with 8.0 μm PET membrane (Corning, New York, NY) was used for invasion assay. The cells (2 × 10^4^) were suspended in 200 μl serum-free medium containing DMSO or Ahr agonists and placed in the trans-well. The lower chamber contained DMSO or Ahr agonists in 750 μl medium supplement with 10 % FBS as an attractant. After 24 h, cells were fixed by formaldehyde and permeabilized with methanol, and then stained with Giemsa. Migrated cells with spread-out shape were counted in 4 different microscopic fields.

### Cell proliferation and adhesion assays

4 × 10^4^ MDA-MB-231 or T47D cells were seeded in 96-well and incubated in 3 % charcoal-stripped FBS DMEM/F-12 containing different concentration of Ahr agonists for 48 h. Cell proliferation was quantified by MTT assay using Cell-Counting Kit-8 (CCK-8; Dojindo, Baltimor, MD) following manufacturer’s instructions. For adhesion assay, 4 × 10^5^ cells were seeded in 6-wells plate and incubated overnight to adhere, then washed to remove non-adherent cells. Cells were then re-incubated in charcoal-stripped medium containing DMSO or Ahr agonists and incubated for 48 h. A single wash was performed before examination.

### Real-time PCR and western blot assays

Extracted RNA was reverse transcribed in a thermal cycler using RT enzyme. The real-time PCR was carried out in ViiA 7 system using TaqMan® gene expression assays. The cycling conditions were 50 °C for 2 min and 95 °C for 10 min, then 40 cycles of 95 °C for 15 s and 60 °C for 1 min. The comparative ΔΔCt method was applied to calculate fold change. For endogenous controls, GAPDH was used for Ahr, CYP1A1 and SOX4, and RNU6B was used for miR-212/132. System, reagents and kits for real-time PCR were all from Applied Biosystems, Grand Island, NY. For western blot, cell were lysed by RIPA lysis buffer system (Santa Cruz Biotechnology), lysate was then fractionated using SDS-PAGE system (Bio-Rad, Richmond, CA). The Ahr, CYP1A1, SOX4 and β-actin were detected using their rabbit polyclonal antibodies (Santa Cruz Biotechnology). The intensities of the protein bands were quantified via software [http://imagej.nih.gov/ij/download.html] ImageJ v.1.48 [52].

### ChIP assay

Analysis of the regulatory elements of the miR-212/132 gene was performed using transcription factor prediction software [http://alggen.lsi.upc.es/] Promo V3.0.2 [31]. The ChIP assay was performed using ChiP-IT enzymatic kit (Active Motif, Carlsbad, CA) following manufacturer’s instructions. Briefly, MDA-MB-231 and T47D cells were treated with DMSO, TCDD or DIM for 24 h. After that Ahr was immunopreciptated using specific antibodies (Santa Cruz Biotechnology). Attached DNA was prepared using proteinase K and further purified using phenol/chloroform procedure. The PCR was done using the following primer sets: XRE-1: forward 5′-CTCCTTCTGCTCCGCGTC-3′, and reverse 5′-TCCGCGGTGCTGATCAAC-3′; XRE-2: forward 5′-AGAGCACTACACCCAGCAG-3′, and reverse 5′-CAGGTGTGAGACTTCCCCAG-3′. The positive control CYP1A1 primers were used as previously described [53]: forward 5′-TCAGGGCTGGGGTCGCAGCGCTTCT-3′, and reverse 5′-GCTACAGCCTACCAGGACTCGGCAG-3′.

### Target gene prediction

Potential binding sites of miR-212/132 cluster (HGNC:31589/HGNC:31516) on the 3′UTR of SOX4 (HGNC:11200) were queried by the miRNA target prediction software microRNA.org [http://www.microrna.org] miRaNda v.21 [32].

### Cell transfection and luciferase reporter assays

The fragment of miR-212/132 promoter that contains XRE-2 was cloned in the Xba1 restriction site of the basic PGL-3 vector (Promega, Madison, WI) using the following primers: forward 5′- AGATCGCCGTGTAATTCTAGAAGAGCACTACACCCAGCAG-3′, and reverse 5′- GCCGGCCGCCCCGACTCTAGACAGGTG TGAGACTTCCCCAG-3′. The siAhr and siNS control (Ambion, Austin, TX) were co-transfected with the miR-212/132 promoter reporter at the final concentration of 75 nmol/L. The 3′UTR-SOX4-luc construct (SwitchGear Genomics, Menlo Park, CA) was co-transfected with miR-212/132 mimics or antisense (Ambion) at 250 nmol/L as previously described [45]. The binding specificity of miR-212/132 cluster on the SOX4 3′UTR was examined by mutation in the sequence on which miR-212/132 seed sequence bind (from GACTGTT to GAGACGG). The MDA-MB-231 and T47D cells were transfected using 4D-nucleofector device and cell-specific transfection kits (Lonza, Walkersville, MD). Cells were incubated for 6 h to recover, and then the medium was changed. After 24 h, the cells were lysed and luciferase activity was measured using luciferase reagents (Promega).

### Orthotopic model of spontaneous metastasis

Female BALB/c athymic nude mice, 6–8 weeks old were purchased from King Faisal Specialist Hospital and Research Center, Riyadh, KSA. The orthotopic model of tumor growth and spontaneous metastasis was induced as previously described using ER-negative and ER-positive breast cancer cells [54]. The MDA-MB-231 and T47D (2.5×10^5^ cells) were suspended in 25 μl of serum-free DMEM. The cells were injected directly into the fat pad of the second left nipple through small incision. The mice receiving T47D cells were implanted with a 90-day release E2 pellets (Innovative Research of America, Sarasota, FL). Non-transplanted (Neg) mice were used as negative controls. The TCDD at 25 μg/kg/day, DIM at 50 mg/kg/day or corn oil were given orally for 10 consecutive days starting from the breast cancer cell injection day. Visible surface tumor nodules and internal nodules were excised under dissection microscope directly after mice euthanization. Lung samples from all mice were fixed in formaldehyde, sectioned to 5 μm thickness and stained with conventional hematoxylin and eosin (H&E) for metastases counting. All animals were maintained under specific pathogen-free conditions and had free access to sterilized feed and water. Animal experiments were performed in accordance with the protocols approved by Animal Care Committee of King Faisal University.

### Statistics

Shown are mean ± SD of the results obtained from three independent experiments studied in triplicates; *n* = 9 in all experiments except when indicated. The significance was analyzed by analysis of variance (ANOVA) test for pooled data from all replicates. *P* < 0.05 was considered significant.

## Additional file

Additional file 1: Figure S1. The role of Ahr in the modulatory effects of TCDD and DIM on migration of MDA-MB-231 and proliferation-based expansion of T47D cells. **Figure S2.** Effects of DIM on proliferation and adhesion of breast cancer cells. **Figure S3.** Effects of TCDD and DIM and two additional Ahr agonists on miR-212/132 expression in breast cancer cells. **Figure S4.** Effects of SOX4 over-expression on the anti-migration, anti-expansion and anti-invasion properties of TCDD and DIM in breast cancer cells. **Figure S5.** Effects of miR-212/132 mimics on mRNA level of SOX4. **Figure S6.** Metastasis model efficiency. (PDF 504 kb)

## Abbreviations

miRNA: MicroRNAs
Ahr: Aryl hydrocarbon receptor
TCDD: 2,3,7,8-tetrachlorodibenzo-p-dioxin
DIM: 3,3′-diindolylmethane
ChIP: Chromatin immunoprecipitation
XRE: Xenobiotic responsive element
3′UTR: 3′ untranslated region
SDC1: Targeting syndecan-1
Runx2: Runt-related transcription factor 2
HN1: Hematological and neurological expressed1
CCH: Columnar cell hyperplasia
ER: Estrogen receptor
MMP: Matrix metalloproteinase
CXCR4: C-X-C chemokine receptor 4
MCDF: 6-methyl-1,3,-trichlorodibenzofuran
HGF: Hepatocyte growth factor
FICZ: 6-formylindolo[3,2-b]carbazole
siAhr: Ahr silencing RNA
siNS: Non-specific nucleotides
ITE: 2-(1′H-indole-3′-carbonyl)-thiazole-4-carboxylic acid methyl ester
3MC: 3-methylcholanthrene
DMEM/F-12: Dulbecco’s modified Eagle’s medium/Ham’s F12 nutrient mixture
as-miR: Antisense hairpin inhibitors
H&E: Hematoxylin and eosin
ANOVA: Analysis of variance
