# Supplementary material for: Aryl hydrocarbon receptor-microRNA-212/132 axis in human breast cancer suppresses metastasis by targeting SOX4
Source: Mol Cancer. 2015 Sep 17;14:172. doi: 10.1186/s12943-015-0443-9 (PMC4573482; doi:10.1186/s12943-015-0443-9)
Supplement: Additional file 1: Figure S1. — The role of Ahr in the modulatory effects of TCDD and DIM on migration of MDA-MB-231 and proliferation-based expansion of T47D cells. Figure S2. Effects of DIM on proliferation and adhesion of breast cancer cells. Figure S3. Effects of TCDD and DIM and two additional Ahr agonists on miR-212/132 expression in breast cancer cells. Figure S4. Effects of SOX4 over-expression on the anti-migration, anti-expansion and anti-invasion properties of TCDD and DIM in breast cancer cells. Figure S5. Effects of miR-212/132 mimics on mRNA level of SOX4. Figure S6. Metastasis model efficiency. (PDF 504 kb) [file 12943_2015_443_MOESM1_ESM.pdf]

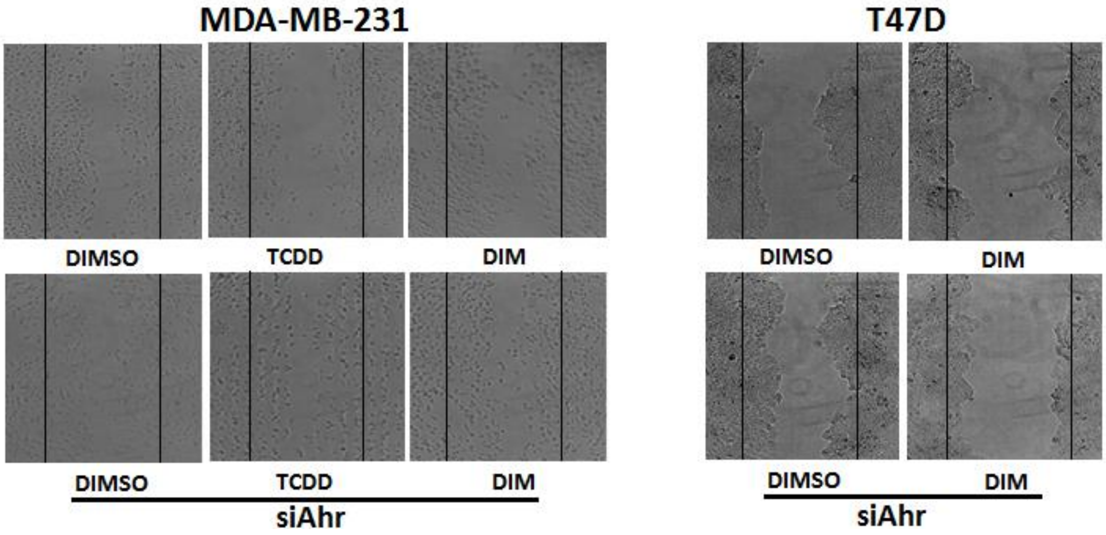


**Figure S1**

Ahr partially mediates the inhibitory effects of DIM on migration of MDA-MB-231 and expansion of T47D. The inhibitory effects of 10 nmol/L TCDD or 25 µmol/L DIM on migration of MDA-MB-231 and expansion of T47D cells were examined by wound healing assay. Inhibition of Ahr using siAhr blocked the effect of TCDD on the migration of MDA-MB-231 cells, while partially inhibited the anti-expansion effect of DIM on both cell lines compared with siNS-transfected control.

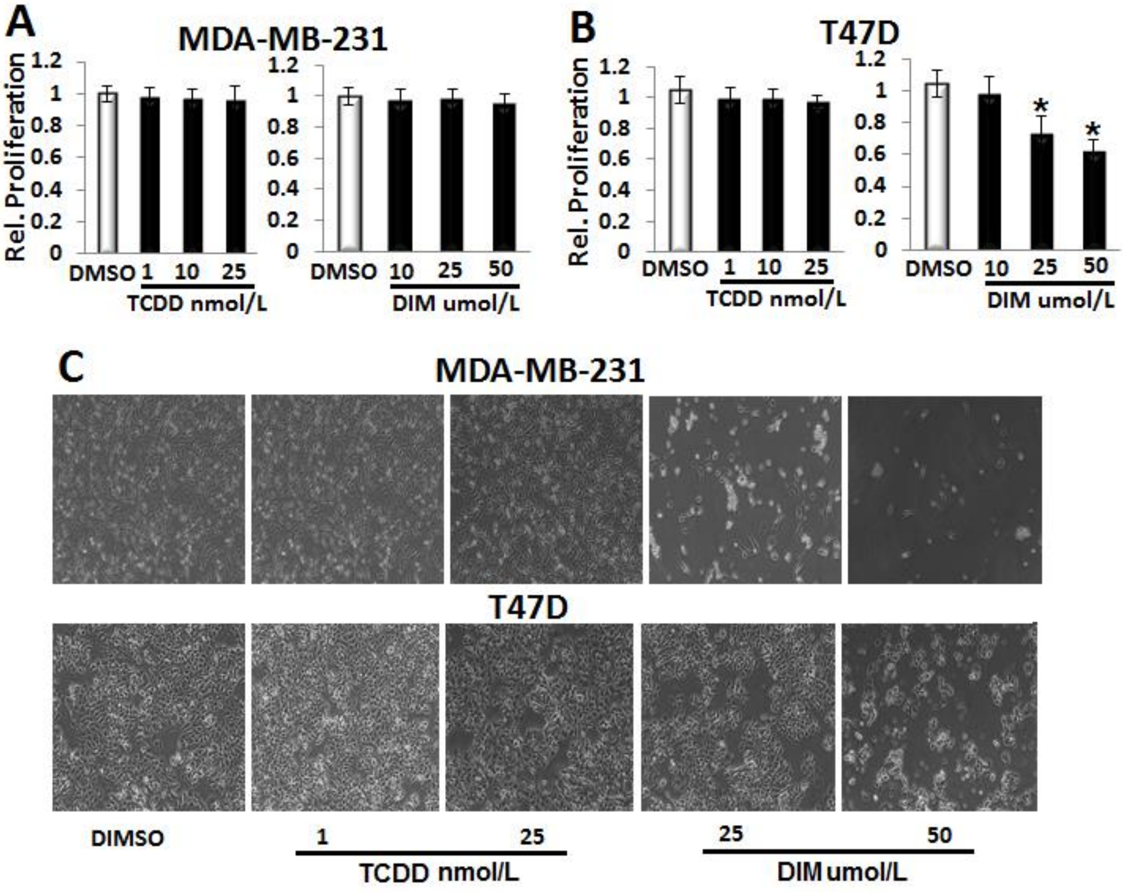


**Figure S2**

DIM suppresses proliferation and adhesion of breast cancer cells. (A and B) Cell proliferation was quantified by MTT assay. Only DIM shows inhibitory effects on proliferation of T47D. (C) DIM suppresses cell adhesion of MDA-MB-231 and T47D cells 48 h after treatment and single PBS wash. Data in A and B are shown as mean ± SD. *; $P<0.05$, significantly different from the DMSO-treated control.

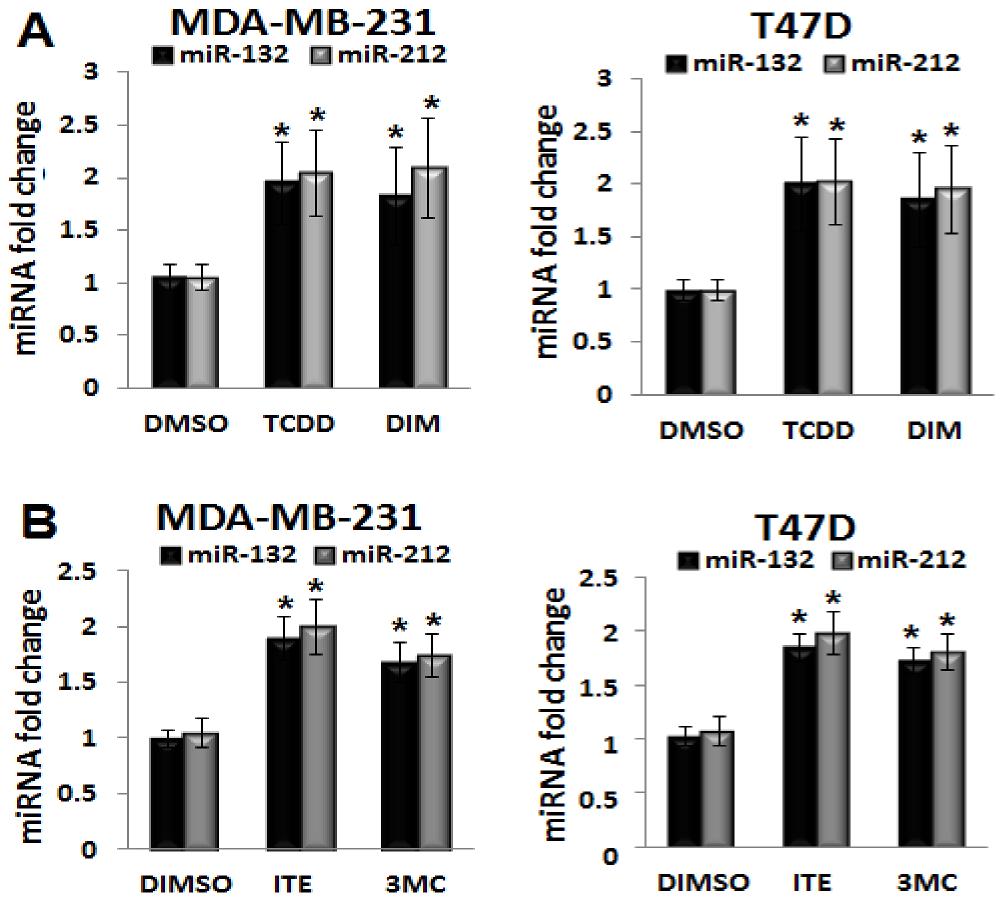


**Figure S3**

Ah agonists induce miR-212/132 cluster in breast cancer cells. (A) TCDD and DIM induces miR-212/132 expression 24 h after treatment. (B) ITE and 3MC induce miR-212/132 transcription 48 h after treatment. Data are shown as mean ± SD. *; $P<0.05$, significantly different from the DMSO-treated control.

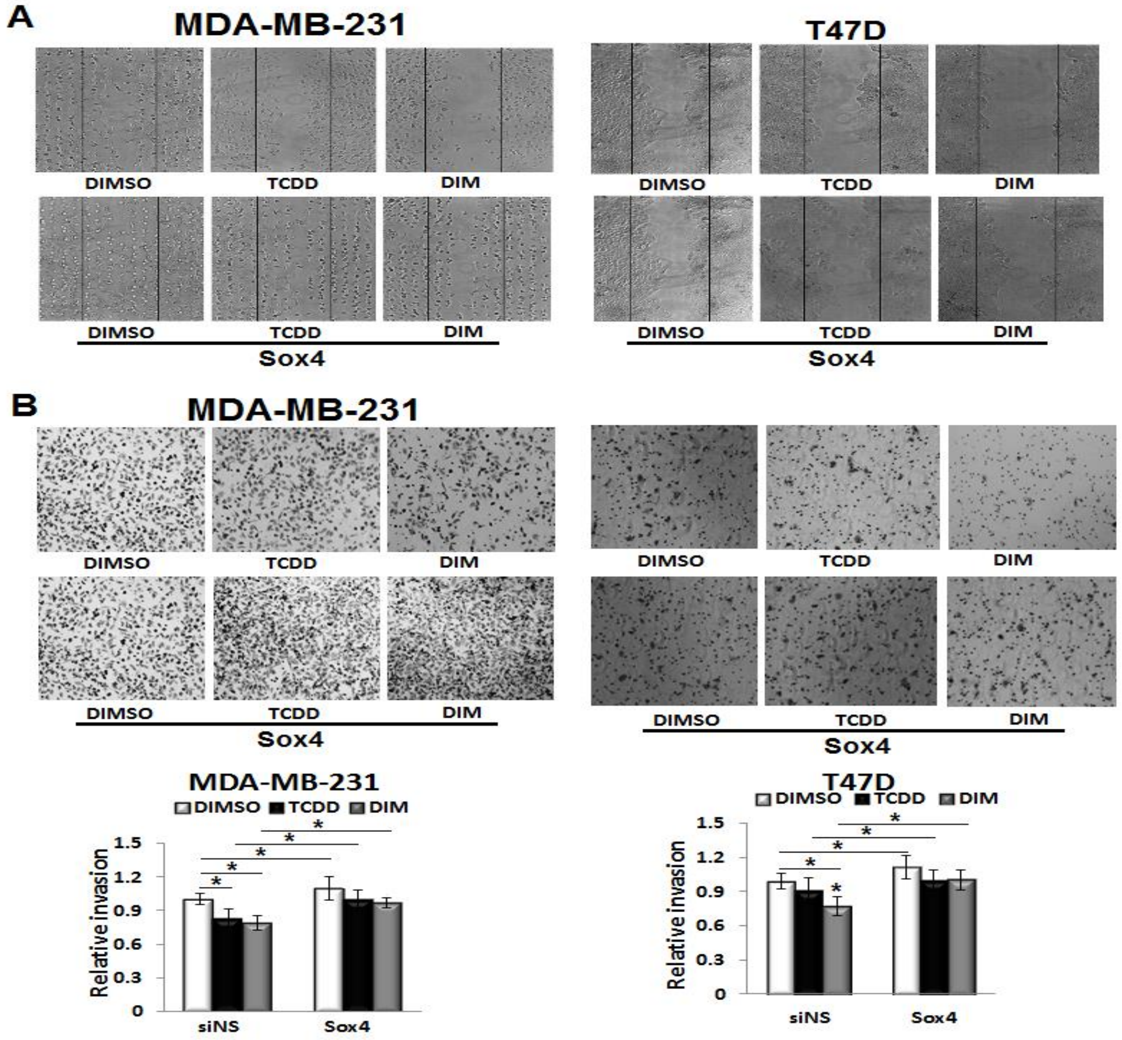


**Figure S4**

The inhibitory effects of Ahr agonists on migration and invasion of breast cancer cells are SOX4-mediated. (A) Over-expression of SOX4 mitigated the inhibitory effects of TCDD and DIM on migration of MDA-MB-231 and T47D cells as examined by wound healing assay. (B) Over-expression of SOX4 mitigated the inhibitory effects of TCDD and DIM on MDA-MB-231 and T47D cells as examined by Boyden chamber assay. Data are shown as mean ± SD. *; $P<0.05$, significantly different from the siNS-transfected control.

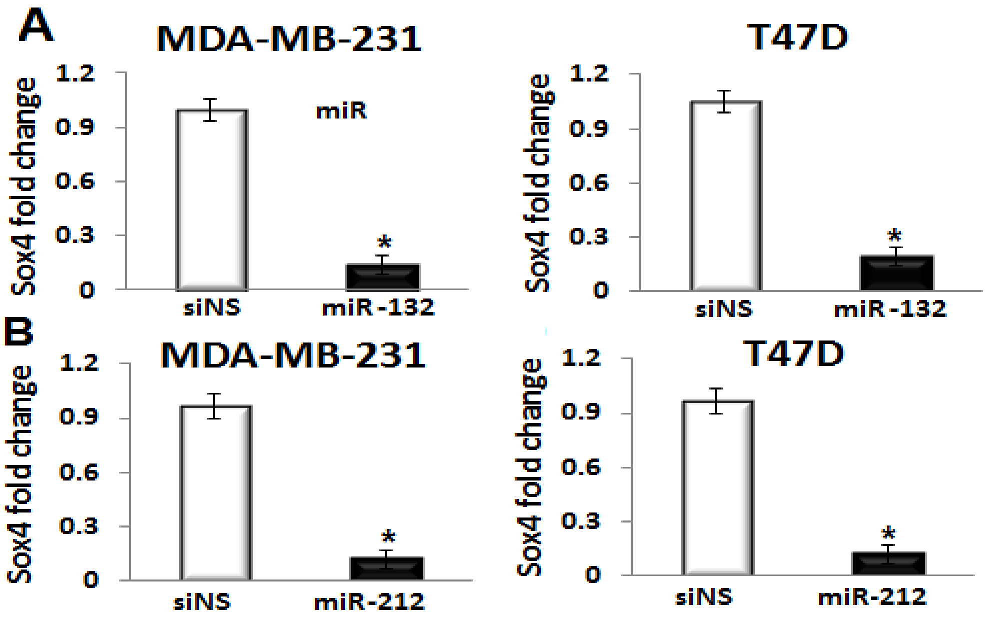


**Figure S5**

Reciprocal correlation between miR-212/132 cluster and SOX4 mRNA in breast cancer cells. MDA-MB-231 and T47D cells were transfected with mimics of (A) miR-132 and (B) miR-212, and SOX4 mRNA was quantified by real-time PCR. Data are shown as mean ± SD. *; P<0.05, significantly different from the siNS-transfected control.

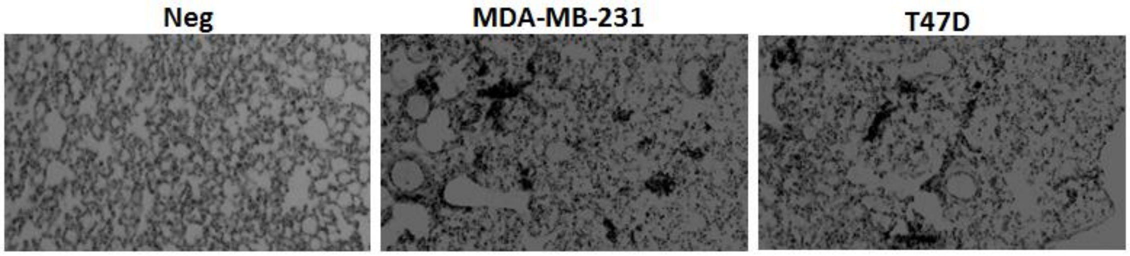


**Figure S6**

Injection of MDA-MB-231 or T47D into the nipple fat pad of athymic mice, resulted in formation of pulmonary nodules that were adequate to represent spontaneous metastasis. Shown are representative H&E staining of the lung metastases from MDA-MB-231- or T47D-injeted mice.
